# Positive Affect Is Inversely Associated with Mortality in Individuals without Depression

**DOI:** 10.3389/fpsyg.2016.01040

**Published:** 2016-07-12

**Authors:** Natalia Martín-María, Francisco Félix Caballero, Beatriz Olaya, Fernando Rodríguez-Artalejo, Josep Maria Haro, Marta Miret, José Luis Ayuso-Mateos

**Affiliations:** ^1^Department of Psychiatry, School of Medicine, Universidad Autónoma de MadridMadrid, Spain; ^2^Department of Psychiatry, Instituto de Investigación Sanitaria Princesa, Hospital Universitario de La PrincesaMadrid, Spain; ^3^CIBER of Mental HealthMadrid, Spain; ^4^Parc Sanitari Sant Joan de DéuBarcelona, Spain; ^5^Department of Preventive Medicine and Public Health, School of Medicine, Universidad Autónoma de Madrid/IdiPAZMadrid, Spain; ^6^CIBER of Epidemiology and Public HealthMadrid, Spain; ^7^Department of Medicine, Universitat de BarcelonaBarcelona, Spain

**Keywords:** experienced well-being, evaluative well-being, mortality, longitudinal study, depression

## Abstract

**Background:** Some studies have analyzed the relation between well-being and mortality but none of them have attempted to disentangle the differential influence that positive affect, negative affect, and evaluative well-being might have on mortality using a longitudinal design in the general population and measuring independently and accurately each component of well-being. The aim of the present study is to assess the association of these well-being components with mortality after adjusting for health and other lifestyle factors and to analyze whether this association is different in people with and without depression.

**Methods:** A nationally representative sample of 4753 people from Spain was followed up after 3 years. Analyses were performed with Cox regression models among the total sample and separately in people with and without depression.

**Results:** In the analyses adjusted for age, sex, and years of education, all three well-being variables showed separately a statistically significant association with mortality. However, after adjustment for health status and other confounders including the other well-being components, only positive affect remained as marginally associated with a decreased risk of mortality in the overall sample [*HR* = 0.87; 95% *CI* = 0.73–1.03], in particular among individuals without depression [*HR* = 0.82; 95% *CI* = 0.68–0.99].

**Conclusion:** Positive affect is inversely associated with mortality in individuals without depression. Future research should focus on assessing interventions associated with a higher level of positive affect.

## Introduction

The determination of individuals' subjective well-being demands a multidimensional assessment including both cognitive judgments as well as affective reactions (Diener, 1984; Ryff and Keyes, 1995). Three aspects of subjective well-being can be distinguished: evaluative well-being (life satisfaction), experienced well-being (the emotions that people experience in their lives), and eudaimonic well-being (sense of purpose and meaning in life).

Recent studies suggest that these three aspects: evaluative well-being (Parker et al., 1992; Lacruz et al., 2011), experienced well-being (Danner et al., 2001; Steptoe and Wardle, 2011), and eudaimonic well-being (Hill and Turiano, 2014; Steptoe et al., 2015) predict lower mortality. In a meta-analysis of prospective studies, Chida and Steptoe (2008) also found that both positive affect and positive dispositions such as life satisfaction, optimism, and sense of humor were associated with significantly reduced mortality. Carstensen et al. (2011) reported that persons who experienced positive emotions more frequently than negative emotions survived longer than those who experienced more negative than positive emotions, and that evaluative well-being also predicted survival. Nevertheless, this result must be generalized with caution, since it come from a relatively small convenience sample of people who at baseline reported having a health status as good as or better than most people their age. Other studies have also examined the effects of life satisfaction, positive affect, and negative affect on mortality, and found that life satisfaction and positive affect predicted mortality (Maier and Smith, 1999; Sadler et al., 2011; Wiest et al., 2011); however, the effect of negative affect was not significant (Maier and Smith, 1999; Wiest et al., 2011). Nevertheless, the instruments used in these studies combined items that measure evaluative well-being (life satisfaction), experienced well-being (positive affect), and some eudaimonic aspects (such as sense of purpose and meaning in life), and were not able to assess the independent impact of each component separately (Tilvis et al., 2012; Wiest et al., 2013; Kern et al., 2014).

Aging increases the positive affect and decreases negative affect (Kahneman and Deaton, 2010; Miret et al., 2014). Evaluative well-being declines with age (Deaton, 2008; Stone et al., 2010), but when the association between age and evaluative well-being is controlled by other variables such as health status, the effect of age disappears (Miret et al., 2014). In contrast, some studies have shown that the decline in health in the last years of life, particularly in people who die after age 85, is accompanied by a decline in well-being. During this terminal decline phase, the correlation between health and well-being becomes stronger (Gerstorf and Ram, 2013).

To our knowledge no longitudinal studies in the general population have yet attempted to disentangle the influence that each aspect of well-being might have on mortality measuring independently and accurately each component and adjusting for health and other lifestyle factors. Therefore, the question of whether experiencing more daily positive emotions, less negative emotions, or judging our lives in a more positive way has a greater mortality impact remains largely unanswered. On the other hand, previous studies have found that the presence of depression is associated with low levels of positive affect (Nutt et al., 2007) and with higher mortality (Bartoli et al., 2013; Gallo et al., 2013). Yet, even though the analyses in some studies have adjusted for depression (Haukkala et al., 2013; Steptoe et al., 2015), it is still uncertain if the association between well-being and mortality is different in people with and without depression. Defining which specific component of well-being has a higher impact on mortality and understanding if the impact is different in people with and without depression, would allow to design targeted interventions that aim at improving a specific aspect of well-being in a specific population.

The aim of the present study was therefore to examine the independent impact of the well-being components (positive affect, negative affect, and evaluative well-being) on mortality after controlling for the effect of a set of established risk factors: health status (Desalvo et al., 2006), multimorbidity (St John et al., 2014), presence of depression (White et al., 2015), body mass index (BMI) (Landi et al., 1999), physical activity (Wen et al., 2011), a healthy diet (Osler and Schroll, 1997), and tobacco (Shaw and Agahi, 2014) and alcohol use (Fichter et al., 2011), and to test whether this impact varies with depression.

## Materials and methods

### Study design and participants

A 3-year longitudinal study was conducted over a nationally representative sample of non-institutionalized adults (aged 18 years or older) from the Spanish population. The baseline data collection was part of the Collaborative Research on Ageing in Europe project (Leonardi et al., 2014).

A multi-stage clustered and age-stratified sampling procedure was employed (less than 50; 50 to 79; 80+). Four strata based on the number of inhabitants of the municipalities were built for each of the 17 Spanish autonomous communities (self-governing regions). Then clusters (census tracts) were selected within the strata with a probability proportional to its size. In each cluster, households were randomly selected, and if there was more than one individual from the corresponding age group in the household, a random method was used to select the individual participant. Interviews were conducted face-to-face at respondents' homes. Participants who were cognitively impaired at baseline did not report data on well-being, and an abridged questionnaire was administered to a proxy interviewee. The number of participants whose information was obtained through a proxy in the first wave of the study was 170 (3.6% of the 4753 who comprised the overall sample). The first wave of the survey was conducted between July 25, 2011 and May 8, 2012; a follow-up was conducted between December 3, 2014 and June 25, 2015. The mean follow-up was 3.25 years (s.d. = 0.18). Quality assurance procedures were implemented during fieldwork (Üstun et al., 2005). Ethical approvals were obtained from the Ethics Review Committees of Hospital Universitario de la Princesa (Madrid) and Parc Sanitari Sant Joan de Déu (Barcelona), as well as written informed consent from participants. The individual response rate was 69.9% at the first wave and 69.5% at the follow-up.

### Measures

Positive affect and negative affect, which are measures of experienced well-being, were assessed using an abbreviated version (Ayuso-Mateos et al., 2013) of the Day Reconstruction Method (DRM) (Kahneman et al., 2004). The abbreviated version of the DRM that has been employed in this study is available at http://www.who.int/healthinfo/systems/GenericIndividualQ.pdf. Participants reconstructed a portion of their previous day's activities, and responded to questions about each episode, including the nature of the activity (e.g., working, shopping) and the extent to which they experienced seven emotions (worried, rushed, irritated, or angry, depressed, tense or stressed, calm or relaxed, and enjoying) on a seven-point response scale ranging from 0 (not at all) to 6 (very much).

Positive affect was defined as the average of the positive emotions (calm/relaxed and enjoying), whereas negative affect was defined as the average of the negative ones (worried, rushed, irritated/angry, depressed, and tense/stressed) (Kahneman et al., 2004). The scores in positive affect and negative affect were weighted by the amount of time people spend in the corresponding activity, therefore, both affect measures are combinations of the duration-weighted affective adjectives that respondents rated for each episode (Krueger and Schkade, 2008). Both scores ranged from 0 to 6, with higher values indicating higher positive affect and higher negative affect, respectively.

Evaluative well-being was measured by means of the Cantril Self-Anchoring Striving Scale (Cantril, 1965), with steps from 0 to 10, in which 0 represents the worst possible life and 10 the best possible life.

Health status was assessed with a set of questions addressing eight health domains: vision, mobility, self-care, cognition, interpersonal activities, pain and discomfort, sleep and energy, and affect (Salomon et al., 2003). For each question, responses were recorded on a 5-point scale, ranging from no difficulty/problem to extreme difficulty/inability. An overall health score was obtained using a Rasch partial credit model (Pallant and Tennant, 2007); it ranged from 0 to 100, with higher scores indicating better health.

Physical multimorbidity was defined as the presence of two or more physical chronic conditions from the following list: chronic lung disease and asthma, diabetes, hypertension, angina pectoris, stroke, and osteoarthritis.

The presence of a depressive episode during the previous 12 months was assessed with a set of questions based on the World Mental Health Survey version of the Composite International Diagnostic Interview (CIDI; Kessler and Üstün, 2004). Individuals were considered to have suffered a depressive episode when they had been diagnosed with depression and had been taking medication or receiving other treatment (e.g., psychotherapy) during the previous 12 months, or when they reported the presence of the core symptoms of the condition during the previous 12 months, according to ICD-10 Diagnostic Criteria for Research (World Health Organization, 1993).

Height and weight were measured with a stadiometer and a calibrated electronic scale, respectively. BMI was calculated as weight in kilograms divided by squared height in meters. Using the standard World Health Organization (WHO) definition, BMI was categorized as < 18.5 kg/m^2^ (underweight), 18.5–24.9 kg/m^2^ (normal weight), and ≥25.0 kg/m^2^ (overweight and obesity) (World Health Organization, 2000).

Physical activity was assessed with the Global Physical Activity Questionnaire version 2 (GPAQ v2) developed by WHO (Bull et al., 2009). Three domains were assessed (activity at work, travel to and from places, and recreational activities), and the responses given by the participants were converted to metabolic equivalent to task (MET) values. The following levels of physical activity were used: low, moderate, and high.

The consumption of vegetables and fruits was collected and categorized as fruit and vegetable (combined) intake of less than vs. 5 or more servings/day.

Tobacco smoking was classified into two categories: current/past daily smokers and never daily smokers. Consumers of at least 5 (in men) or 4 (in women) standard alcoholic drinks per day on at least 1 day in the week before the interview were considered “heavy” drinkers. Those who had ever consumed alcohol but were not heavy drinkers were categorized as “non-heavy” drinkers (World Health Organization, 2002).

Participants were also asked to provide sociodemographic information: age, sex, current marital status, years of education, residential setting, and quintile of household income.

Mortality was ascertained just before the follow-up evaluation through the National Death Index, which contains information on the vital status of all the residents in Spain. Since the register is updated with some months of delay, information on additional participants who had passed away was obtained during the household visits in the follow-up assessment.

### Statistical analysis

All data were weighted to account for sampling design and to generalize the study sample to the Spanish population. Normalized weights were employed. Post-stratification corrections were made to the weights to adjust for the population distribution obtained from the national census and for non-response (Moussavi et al., 2007). Frequency analysis and descriptive statistics were used to summarize the characteristics of the sample. Differences in socio-demographics and well-being variables between participants who died during the follow-up and participants who were alive at the end of follow-up were tested by unpaired *t*-tests and χ^2^-tests, for quantitative and categorical variables, respectively. Hedges' *g* (unpaired *t*-test) and Cramer's *V* (χ^2^-test) were reported as effect size measures in case of significant differences at the 95% confidence level. Cohen's guidelines (Cohen, 1988) were used as the standard to evaluate the magnitude of effect size: Hedges' *g* values of 0.20, 0.50, and 0.80, constitute small, medium, and large effect sizes; these values were 0.10, 0.30, and 0.50, respectively, for Cramer's *V*.

A two-step procedure based on the Cox proportional hazards regression model was used to assess the association of well-being and the rest of study variables with mortality. In the first step, a model was built for each of the variables described in the *Measures* section, with adjustment for age, sex, and years of education. In the second step, all variables with a *p* < 0.1 (a conservative threshold) were included in a subsequent multivariate model, which also controlled for age, sex, and years of education. Hazard ratios (HR) and their 95% confidence interval (CI) were used to summarize the study associations. Interaction terms for well-being variables and depression were included to assess whether the relationship between well-being and mortality varied with depression. Separate analyses were also conducted in persons with depression and without; survival curves were generated using Kaplan-Meier estimates and the log-rank test was used to compare those curves according to depression status.

In addition, the analyses were replicated among participants who survived for at least 3 months, to rule out the role of pre-existing poor health status leading to diminished well-being and premature mortality. Analyses were carried out using Stata SE version 11 (Statacorp, 2010).

## Results

A total of 259 participants died during the follow-up (5.44% of the total sample comprising 4753 participants). Of these, 25 participants (9.70% of the deceased participants) died during the first 90 days of the follow-up. The number of people who died and had reported data on well-being at baseline was 179 (15 participants died in the first 90 days of the follow-up). The remaining 80 participants who died were unable to participate in the baseline interview by themselves due to their poor health status, so their information was obtained through a proxy respondent who did not respond to the subjective well-being questions.

Baseline characteristics of individuals who died or remained alive during the follow-up are shown in Table 1. The mean age of the deceased participants was 77.95 years (s.d. = 11.60), with 44.0% being women. Socio-demographics are also shown in Table 1. According to the effect sizes, the main differences between people who survived and people who died during the follow-up were found for age and years of education.

**Table 1:** **Baseline characteristics of study participants according to survival status at the end of follow-up**.

	**People who survived (*n* = 4494)**	**People who died (*n* = 259)**	***t*/χ^2^ (d.f.)**	***p***	**Effect size**
**SOCIO-DEMOGRAPHICS**					
Female: *n* (%)	2488 (55.4)	114 (44.0)	12.73 (1)	< 0.001	0.05
Age, years: Mean ± s.d.	59.43 ± 15.89	77.95 ± 11.60	18.47 (4751)	< 0.001	1.18
Years of education: Mean ± s.d.	10.96 ± 6.25	7.42 ± 5.86	7.41 (4555)	< 0.001	0.57
Married or in partnership: *n* (%)	2720 (60.5)	125 (48.3)	15.33 (1)	< 0.001	0.06
Rural setting: *n* (%)	620 (13.8)	38 (14.7)	0.16 (1)	0.69	–
First or second quintile of household income: *n* (%)	1599 (40.3)	76 (46.3)	2.42 (1)	0.12	–
**MEAN ESTIMATES (95% CI)**					
Positive affect	4.85 (4.80–4.91)	4.88 (4.66–5.10)	0.25 (4582)	0.81	–
Negative affect	0.67 (0.63–0.71)	0.70 (0.54–0.86)	0.33 (4582)	0.75	–
Evaluative well-being	6.78 (6.71–6.86)	6.00 (5.71–6.29)	5.09 (4555)	< 0.001	0.47

Hedges' g for unpaired t-tests (quantitative variables) and Cramer's V for χ^2^-tests (categorical variables) were considered as effect size measures for statistically significant differences. d.f., Degrees of freedom.

Regarding the well-being variables, mean values are also reported in Table 1. Significantly higher scores on evaluative well-being were found for people who survived [*t*_(1)_ = 5.09; *p* < 0.001, Hedges' *g* = 0.47], although this first analysis was conducted without taking into account the elapsed time until death or controlling for other covariates. The seven emotions reported in the DRM, and used for generating positive and negative affect measures, were separately considered in a descriptive analysis over the total sample. Mean scores were calculated considering the average for each participant in each item, and higher scores indicated a higher perception of the corresponding emotion. The scores ranged between 0 and 6 in the seven items. Similar means were found across the negative items, with the following mean ± s.d.: 0.59 ± 0.92 for worried, 0.66 ± 0.95 for rushed, 0.52 ± 0.83 for irritated/angry, 0.61 ± 1.02 for depressed, and 0.67 ± 0.99 for tense/stressed. In the case of the two positive items, similar mean values were found: 4.88 ± 1.13 for calm/relaxed and 4.96 ± 1.06 for enjoying.

The three well-being variables (positive affect, negative affect and evaluative well-being) showed a significant association with mortality in the analyses conducted over the overall sample, after adjusting for age, sex, and years of education (Table 2). However, after additional control for other potential confounders, only positive affect remained marginally associated; specifically, positive affect was linked to lower mortality (*HR* = 0.87; 95% *CI* = 0.73–1.03, *p* = 0.09) in the final model.

**Table 2:** **Hazard ratios (HR) and their 95% confidence interval (CI) for mortality according to well-being and other variables**.

	**Adjusted for age, sex, and years of education**			**Final model**		
	**HR**	**95% CI**	***p***	**HR**	**95% CI**	***p***
Positive affect	0.85	0.75–0.97	0.012	0.87	0.73–1.03	0.09
Negative affect	1.17	1.01–1.37	0.047	0.98	0.81–1.19	0.83
Evaluative well-being	0.84	0.77–0.91	< 0.001	1.05	0.93–1.16	0.46
Health status	0.94	0.92–0.95	< 0.001	0.95	0.93–0.97	< 0.001
Physical multimorbidity (ref. No)	1.32	0.97–1.79	0.08	0.82	0.57–1.19	0.29
Body mass index (ref. normal)						
Underweight	6.93	2.45–19.63	< 0.001	5.47	1.88–15.94	0.002
Overweight/obesity	0.72	0.48–1.06	0.09	0.70	0.47–1.04	0.08
Physical activity (ref. high)						
Moderate	1.48	0.93–2.36	0.10	1.40	0.87–2.25	0.17
Low	2.40	1.54–3.74	< 0.001	1.28	0.78–2.09	0.33
Daily fruit and vegetable intake (ref. less than 5 servings)	0.82	0.58–1.16	0.26	–	–	–
Tobacco consumption (ref. never smoker)	1.44	1.02–2.03	0.040	1.31	0.90–1.93	0.16
Heavy alcohol consumption (ref. No)	0.28	0.04–1.99	0.21	–	–	–
Married or in partnership (ref. No)	0.73	0.52–1.01	0.06	0.84	0.58–1.21	0.35
Residential setting (ref. rural)	1.17	0.75–1.84	0.49	–	–	–
Household income (ref. first or second quintile of income)	0.94	0.68–1.29	0.70	–	–	–
Sex (ref. male)	–	–	–	0.34	0.23–0.51	< 0.001
Age	–	–	–	1.08	1.07–1.10	< 0.001
Years of education	–	–	–	0.98	0.95–1.01	0.25

A first model assessed the association of each variable with mortality, adjusting only for age, sex and years of education. The final model additionally included the covariates with a p-value < 0.1 in the first model. Positive affect and negative affect scores ranged between 0 and 6, with higher scores indicating higher positive affect and negative affect, respectively. Evaluative well-being ranged between 0 and 10, with higher scores indicating higher evaluative well-being. The HR corresponding to the three well-being variables indicates the increase/decrease in the risk of mortality per one unit increased.

**Figure 1:**
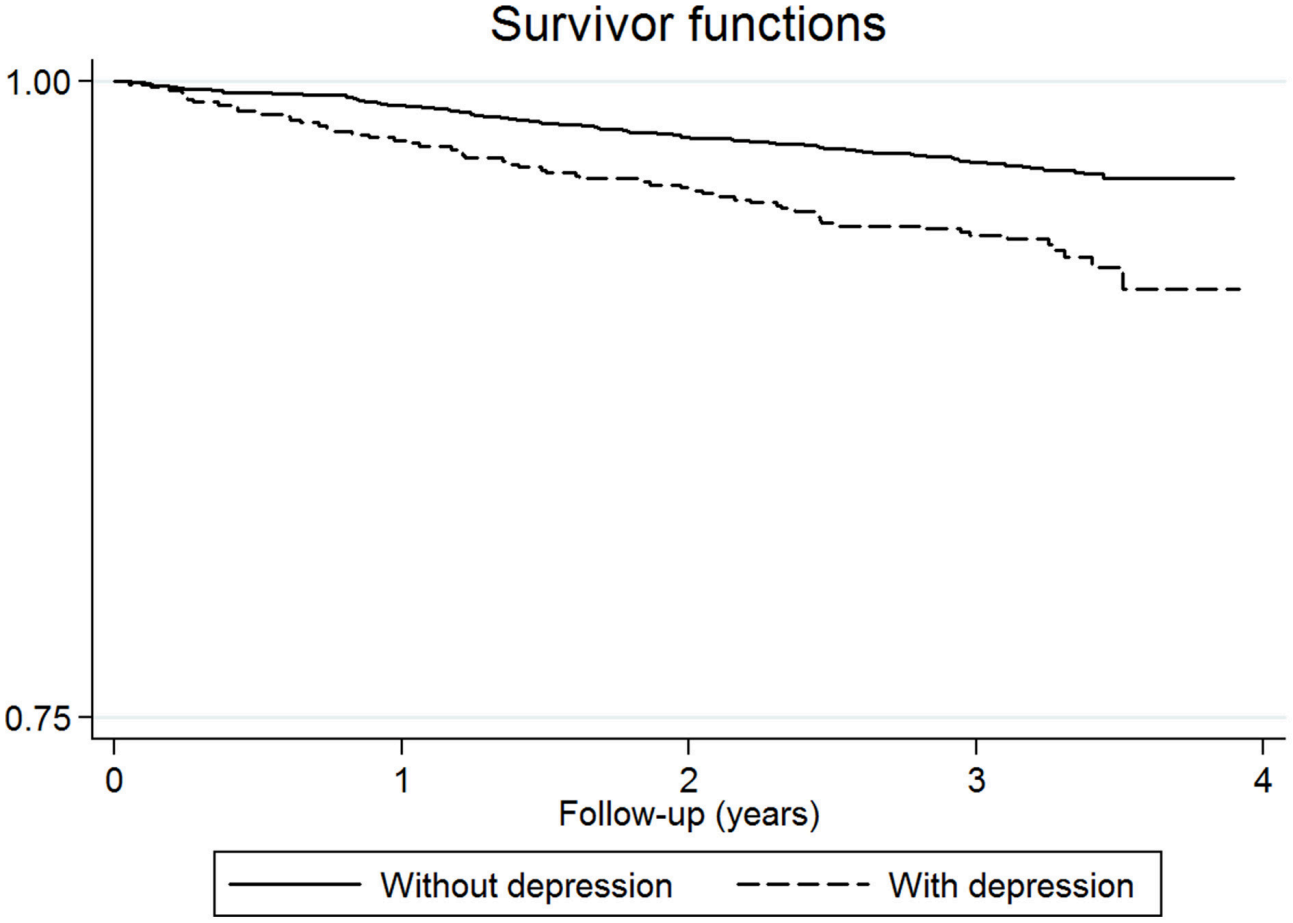
**Proportion of individuals with and without depression surviving over the follow-up period**.

A total of 863 participants presented depression at baseline, and 60 (6.95%) of them died during the follow-up. Before assessing the relationship between the well-being variables and mortality separately in participants with and without depression, the interaction terms between well-being variables and depression were included in the Cox proportional hazards regression model above-mentioned. The results associated to the three interaction terms were not significant although a slight trend was observed in the interaction between positive affect and depression [*HR* = 1.32; 95% *CI* = (0.82, 2.10), *p* = 0.25]. On the other hand, the log-rank test showed that the survival for people with and without depression was different over time [$\chi_{\left(1\right)}^{2}$ = 18.99, *p* < 0.001] (Figure 1). The analyses based on the presence of depression are shown in Table 3, where the HRs, 95% CI, and *p*-values associated to the relationships between the well-being variables and mortality in the two groups are provided, and where the coefficients associated to the remaining covariates considered are also reported. In people without depression, positive affect was found to be associated with a decreased risk of mortality (*HR* = 0.82; 95% *CI* = 0.68–0.99, *p* = 0.038).

**Table 3:** **Hazard ratios (HR) and their 95% confidence interval (CI) for mortality in people who had depression and people who did not have depression**.

	**With depression (*****n*** = **736)**			**Without depression (*****n*** = **3561)**		
	**HR**	**95% CI**	***p***	**HR**	**95% CI**	***p***
Positive affect	1.13	0.74–1.72	0.58	0.82	0.68–0.99	0.038
Negative affect	0.95	0.63–1.43	0.81	1.08	0.85–1.37	0.54
Evaluative well-being	1.00	0.79–1.27	0.99	1.05	0.93–1.20	0.42
Health status	0.97	0.92–1.02	0.23	0.95	0.93–0.98	0.001
Physical multimorbidity (ref. No)	0.75	0.32–1.75	0.50	0.84	0.56–1.27	0.41
Body mass index (ref. normal)						
Underweight	9.78	1.94–49.21	0.006	2.04	0.27–15.40	0.49
Overweight/obesity	0.55	0.22–1.40	0.21	0.75	0.48–1.16	0.20
Physical activity (ref. high)						
Moderate	1.40	0.41–4.78	0.60	1.39	0.83–2.35	0.21
Low	2.00	0.60–6.60	0.28	1.16	0.67–2.00	0.59
Tobacco consumption (ref. never smoker)	0.94	0.37–2.41	0.90	1.30	0.85–2.00	0.23
Married or in partnership (ref. No)	1.24	0.56–2.76	0.60	1.19	0.78–1.82	0.41
Sex (ref. male)	0.19	0.08–0.45	< 0.001	0.40	0.25–0.64	< 0.001
Age	1.05	1.01–1.08	0.011	1.09	1.07–1.12	< 0.001
Years of education	0.98	0.90–1.08	0.52	0.98	0.95–1.02	0.34

Well-being variables and covariates considered in the final Cox regression model for the total sample were included as independent variables in both models. Positive affect and negative affect scores ranged between 0 and 6, with higher scores indicating higher positive affect and negative affect, respectively. Evaluative well-being ranged between 0 and 10, with higher scores indicating higher evaluative well-being. The HR corresponding to the three well-being variables indicates the increase/decrease in the risk of mortality per 1 unit increased.

The analyses were also run with 4178 individuals who survived for at least 3 months, obtaining similar results. Positive affect continued to show a lower risk of death among those without depression (*n* = 3469) (*HR* = 0.82; 95% *CI* = 0.67–0.99, *p* = 0.049).

## Discussion

This study found that experiencing positive emotions is associated with lower risk of death, after controlling for other well-being components and potential confounders, in a representative sample of the Spanish general population.

These results are congruent with the results of some previous studies (Danner et al., 2001; Steptoe and Wardle, 2011), in which the protective effect of experienced positive affect, even over a single day, had a stronger relationship with mortality, not explainable only by health status or sociodemographic characteristics. In contrast, Liu et al. (2015) found that the effect that experienced well-being had on mortality disappeared after adjusting for self-rated health. These different results might be due to the different instrument used to assess experienced well-being. Whereas, Liu et al. (2015) relied on a single question on the frequency of feeling happiness, in our study an abbreviated version (Ayuso-Mateos et al., 2013) of the DRM (Kahneman et al., 2004) was used. By asking respondents to reconstruct the previous day by completing a structured questionnaire, the DRM results in a lower susceptibility to memory and judgmental biases than global reports of daily experiences (Kahneman et al., 2004; Oishi, 2010).

Both experienced and evaluative well-being were associated with lower risk of mortality after adjusting for age, sex, and years of education. However, after additionally controlling for other confounders, such as health status, only positive affect remained to be marginally associated with a decreased risk of mortality. The fact that the relationship between evaluative well-being and mortality disappeared after controlling for health status might be due to the strong correlation between health status and evaluative well-being. Comparing positive and negative affect, the results indicate that positive affect is a stronger predictor of mortality than negative affect. In fact, in line with other studies (Maier and Smith, 1999; Wiest et al., 2011), the impact of negative affect was not significant.

Moreover, the results showed that positive affect was related to a low risk of mortality in participants without depression, but not in people with depression. Previous studies have found that the presence of depression is associated with low levels of positive affect (Nutt et al., 2007) and with higher mortality (Bartoli et al., 2013; Gallo et al., 2013). These results may suggest that experiencing positive affect is not enough to reduce the mortality of people with depression. The fact that the relation of each component of well-being diminished when other health-related variables and the components of well-being were introduced into the same model, can be due to the correlation between all the components (Kahneman and Deaton, 2010) and the adjustment for covariates as health status, which is strongly associated with well-being (Miret et al., 2014).

To our knowledge, this is the first study to analyze longitudinally the different impact of positive affect, negative affect, and evaluative well-being on mortality in a large representative sample of the general population covering a wide age range, controlling for the most relevant covariates as well as well-being variables. Moreover, the similar trend found when the analysis was run excluding participants who died in the following three months after the baseline interview, indicates that the association might not be completely explained by the fact that the terminal decline in health lead both to terminal decline in well-being and premature mortality (Gerstorf and Ram, 2013).

The results of this study should be interpreted taking into account some limitations. Eudaimonic well-being was not evaluated. Since previous studies have found an association of purpose in life (Hill and Turiano, 2014) and sense of coherence (Haukkala et al., 2013) with mortality, future research should also include this component. Although the analyses were controlled by health status at baseline, reversed causality cannot be completely ruled out. Another limitation was the short follow-up period, which implied a moderately small number of participants who presented the event (death) at the end of the follow-up.

The results of this study suggest that it would be advisable to explore the affective experience of patients in order to detect those with low positive affect, since interventions to improve well-being, specifically those focused on promoting activities that are associated with a higher level of positive affect, might lower mortality. Encouraging people to engage in some kind of exercise and to participate in recreational and social activities might raise their experience of happiness, helping them to live longer and in a healthier way. These findings also support the value of assessing experienced well-being, and the importance of evaluating interventions that promote positive emotions in the general population. Periodic and longitudinal assessments of a variety of measures of subjective well-being over time should confirm our results.

## Author contributions

FC, BO, JH, MM, and JA designed the study and oversaw all aspects of the study implementation and data collection. NM, FC, and MM wrote the first draft. BO, FR, JH, and JA reviewed the first draft of the manuscript and made substantial contributions to the interpretation of the data. NM and FC carried out the statistical analyses. NM, FC, MM, and JA conceptualized and oversaw analyses, and made substantial contributions to the interpretation of data. All authors made critical revision of the final draft and agree to be accountable for all aspects of the work in ensuring that questions related to the accuracy or integrity of any part of the work are appropriately investigated and resolved.

## Funding

The research leading to these results has received funding from the European Union Horizon 2020 Framework Programme for Research and Innovation under grant agreement 635316 (ATHLOS Project), from the European Community's Seventh Framework Programme (FP7/2007-2013) under grant agreement number 223071 (COURAGE in Europe), from the Spanish Ministry of Science and Innovation ACI-Promociona (ACI2009-1010), from the Centro de Investigación Biomédica en Red de Salud Mental (CIBERSAM) Mental Health and Disability Instruments Library Platform, and from the Instituto de Salud Carlos III-FIS research grants PS09/00295, PS09/01845, PI12/01490, and PI13/00059. Projects PI12/01490 and PI13/00059 have been co-funded by the European Union European Regional Development Fund (ERDF) “A Way to Build Europe.” The study was supported by the Instituto de Salud Carlos III Centro de Investigación Biomédica en Red de Salud Mental (CIBERSAM). NMM is supported by the programme “Contratos predoctorales para Formación de Personal Investigador, FPI-UAM,” Universidad Autónoma de Madrid, Spain.

### Conflict of interest statement

The authors declare that the research was conducted in the absence of any commercial or financial relationships that could be construed as a potential conflict of interest.
